# Long-term survival of patients with stage III colon cancer treated with VRP-CEA(6D), an alphavirus vector that increases the CD8+ effector memory T cell to Treg ratio

**DOI:** 10.1136/jitc-2020-001662

**Published:** 2020-11-11

**Authors:** Erika J Crosby, Amy C Hobeika, Donna Niedzwiecki, Christel Rushing, David Hsu, Peter Berglund, Jonathan Smith, Takuya Osada, William R Gwin III, Zachary C Hartman, Michael A Morse, Herbert Kim Lyerly

**Affiliations:** 1 Surgery, Duke University School of Medicine, Durham, North Carolina, USA; 2 Biostatistics and Bioinformatics, Duke University School of Medicine, Durham, North Carolina, USA; 3 Biostatistics, Duke Cancer Institute, Durham, North Carolina, USA; 4 Medicine, Duke University School of Medicine, Durham, North Carolina, USA; 5 HDT Bio Corp, Seattle, Washington, USA; 6 VLP Therapeutics, Gaithersburg, Maryland, USA; 7 Medicine, University of Washington, Seattle, Washington, USA; 8 Pathology, Duke University School of Medicine, Durham, North Carolina, USA; 9 Immunology, Duke University School of Medicine, Durham, North Carolina, USA

**Keywords:** CD4-CD8 Ratio, clinical trials as topic, immunotherapy, vaccination, adaptive immunity

## Abstract

**Background:**

There remains a significant need to eliminate the risk of recurrence of resected cancers. Cancer vaccines are well tolerated and activate tumor-specific immune effectors and lead to long-term survival in some patients. We hypothesized that vaccination with alphaviral replicon particles encoding tumor associated antigens would generate clinically significant antitumor immunity to enable prolonged overall survival (OS) in patients with both metastatic and resected cancer.

**Methods:**

OS was monitored for patients with stage IV cancer treated in a phase I study of virus-like replicon particle (VRP)-carcinoembryonic antigen (CEA), an alphaviral replicon particle encoding a modified CEA. An expansion cohort of patients (n=12) with resected stage III colorectal cancer who had completed their standard postoperative adjuvant chemotherapy was administered VRP-CEA every 3 weeks for a total of 4 immunizations. OS and relapse-free survival (RFS) were determined, as well as preimmunization and postimmunization cellular and humoral immunity.

**Results:**

Among the patients with stage IV cancer, median follow-up was 10.9 years and 5-year survival was 17%, (95% CI 6% to 33%). Among the patients with stage III cancer, the 5-year RFS was 75%, (95%CI 40% to 91%); no deaths were observed. At a median follow-up of 5.8 years (range: 3.9–7.0 years) all patients were still alive. All patients demonstrated CEA-specific humoral immunity. Patients with stage III cancer had an increase in CD8 +T_EM_ (in 10/12) and decrease in FOXP3 +Tregs (in 10/12) following vaccination. Further, CEA-specific, IFNγ-producing CD8+granzyme B+T_CM_ cells were increased.

**Conclusions:**

VRP-CEA induces antigen-specific effector T cells while decreasing Tregs, suggesting favorable immune modulation. Long-term survivors were identified in both cohorts, suggesting the OS may be prolonged.

## Background

Colon cancer, while curable with surgery alone when localized to the bowel wall, has high rates of relapse when metastatic to lymph nodes (stage III) and high mortality rates when distantly metastatic (stage IV).^1^ Chemotherapy for stage III^2 3^ and chemotherapy plus biologic therapy with VEGF and EGFR-targeted antibodies in stage IV^4^ can both improve survival, but additional therapies are needed to extend these survival benefits.

Infiltration of colon cancers by CD8+T cells is associated with improved recurrence free survival,^5^ which suggests that attempts to augment the immune response against colon cancer may be beneficial. The success of immune checkpoint blockade (ICB) in metastatic microsatellite instability (MSI) high colorectal cancer^6^ has increased interest in immunotherapy for the remaining 85%–96% of patients with MSI; however, ICB alone has been ineffective in this subgroup. Mechanistically, the neoantigens generated by the genetic instability of MSI patients is thought to increase the number of potential tumor-specific effector T cells, which can be activated by ICB. Because more than 80% of colon cancer is MSI negative,^7^ alternative strategies to increase infiltrating T cells (TIL) and subsequent responses to ICB are needed.

Cancer vaccines that activate immune responses against tumor-expressed antigens, may be an option for increasing TIL and extending the efficacy of immunotherapy to colorectal cancer.^8^ Colon cancers express several defined antigens, relatively restricted to the tumor and known to be targets of immune effectors, with carcinoembryonic antigen (CEA) being one of the most extensively studied.^9^ Although studies frequently report induction of immune response against target antigens, clinical benefit associated with immunizations in the absence of ICB has been modest.^10^ Two potential explanations are the use of vaccines in heavily pretreated advanced cancer patients and immunization platforms that simultaneously activate immunosuppressive regulatory T cells along with the desired effector T cell response.^11^

Vaccine platforms for targeting CEA have included peptides, proteins, modified tumor cells, DNA, mRNA, viral vectors, and dendritic cells (DC).^10^ We previously reported on the use of virus-like replicon particle (VRP)-CEA (AVX701), an alpha-VRP, based on attenuated Venezuelan equine encephalitis virus encoding the modified epitope CEA(6D) which we designate as VRP-CEA, to activate CEA-specific immune responses.^12^ CEA(6D) refers to an Asn to Asp substitution in CEA which results in enhanced recognition by cognate CD8+T cell receptors.^13^ Advantages of the VRP vaccine platform include their tropism for professional antigen-presenting cells (DCs), the capacity to replace their structural gene region with foreign genes, and the production of self-replicating RNA transcripts, resulting in generation of large amounts of the encoded heterologous proteins and induction of potent cellular and humoral immunity against these proteins.^14^ In the phase I clinical trial of VRP-CEA enrolling heavily pretreated patients with metastatic colorectal cancer,^12^ there was no dose limiting toxicity and the highest dose tested (4×10^8^ IU) was determined to be the maximal feasible dose. CEA-specific T cell and antibody responses following VRP-CEA vaccination were observed regardless of the development of antiviral neutralizing antibodies or regulatory T cell frequency.

We hypothesized that generating a high frequency of circulating CEA specific T cells generated, even with increased regulatory T cells, would provide some clinical benefit. Using a complex DC and pox vector based cancer vaccine, we have previously demonstrated improved survival of patients with metastatic colorectal cancer following metastasectomy and administration of the vaccine.^15^ These findings suggest that vaccination in a clinical scenario with minimal tumor-induced immunosuppression may be more effective, even without ICB. For these reasons, it has been suggested that patients following tumor resection with no evidence of residual disease but at a high risk of relapse may benefit from a vaccine that induces an adaptive immune response and could offer an improved overall survival (OS).

Consequently, we monitored the long-term survival of all patients with stage IV cancer who had been vaccinated with VRP-CEA. We observed three long-term overall survivors of over 10 years in patients with stage IV cancer that were vaccinated and underwent subsequent tumor debulking. We then enrolled an expansion cohort of patients with stage III colon cancer following surgery and completion of adjuvant systemic chemotherapy. At a median follow-up of 5.8 years (range: 3.9–7.0 years), a 100% OS and 85% PFS among the patients with stage III colon cancer were vaccinated. Further, we identified increases in antigen-specific effector T cell responses and a reduction in Treg as early as after one vaccination.

## Methods

### Patients with stage III cancer and study drug administration

All patients were enrolled and treated under an FDA-approved Investigational New Drug Exemption and registered at ClinicalTrials.gov (NCT00529984, NCT01890213). Participants were recruited from medical oncology clinics at Duke University Medical Center, Durham, North Carolina, USA and provided consent under protocols approved by the Duke University Medical Center Institutional Review Board (Pro00045976). Eligibility requirements for the stage IV study were previously reported.^12^ Eligibility requirements for the stage III study included age >18, histologically confirmed stage III colon cancer as determined by the AJCC 7th edition, and receipt of adjuvant postoperative chemotherapy (5-fluorouracil-based regimen with or without oxaliplatin for at least six cycles or capecitabine with or without oxaliplatin for four cycles). Chemotherapy must have been completed 1–6 months before initiating study treatment. Other requirements were ECOG status of 0 or 1, adequate hematologic counts, and hepatic and renal function. Known autoimmune disease or HIV infection, concurrent immunosuppressive therapies, and significant cardiovascular disease or arrhythmias were exclusionary criteria.

VRP-CEA was administered at 4×10^8^ IU intramuscularly into the deltoid (alternating arms with each injection) every 3 weeks for four administrations. Chest, abdominal, and pelvic CT or MRI scans and serum CEA level were performed as part of a patient’s standard management at baseline and after the final vaccination. Blood samples were taken pretreatment, prior to each injection (weeks 0, 3, 6, and 9), and 3 weeks post-treatment. Patients or their physicians were contacted approximately every 6 months for survival and progression status after completing study immunizations

### CYTOF flow cytometry analysis

PBMC stimulation: Frozen vials were thawed and rested overnight at 37°C. The timepoint postvaccination for each patient was chosen based on availability of PMBC samples (one patient at week 3, three patients at week 6, five patients at week 9, and two patients at week 12). Cells were washed with warm RPMI-1640 medium (Hyclone) supplemented with 10% fetal bovine serum, (FBS) (Atlanta Biologicals), 100 U/mL penicillin, 100 µg/mL streptomycin, 29.2 mg/mL L-glutamine (Hyclone) and 25 U/mL benzonase (Sigma-Aldrich) then resuspended at 5×10^6^ cells/mL. Cells were restimulated with BFA/monensin (1× both eBioscience) alone (negative control), BFA/monensin/PMA (500 ng/mL, Sigma)/Ionomycin (1 µg/mL, Sigma) (positive control), or BFA/monensin/TRICOM-CEA^16^ (10 MOI/cell; Lot#MFG-72299, a different vector expressing CEA to stimulated CEA but not VRP specific cells) for 5 hours prior to cytometry time of flight (CYTOF) staining.

CYTOF antibody panel (online supplemental table 1) lists the 28 labels with the corresponding antibodies. Antibodies were purchased from Fluidigm; those in gray were purchased from the Lederer lab at the Brigham and Women’s Hospital; CTLA4-FITC was purchased from ThermoFisher. Concentration of each antibody was titrated and optimized individually.

10.1136/jitc-2020-001662.supp1 Supplementary data

Mass cytometry: Staining of samples was adapted from Protocol PN 400279 A4 (Fluidigm). Briefly, cells were then stained with Cell-ID Cisplatin at a final concentration of 1 µM before FC blocking (TruStain FcX, Biolegend). Control PBMCs were stained with CD45-115In and experimental samples were stained with CD45-89Y in cell staining buffer (Fluidigm). Control PBMCs were spiked into each sample for a final proportion of 20% control cells: 80% experimental. Cells were stained with the remaining surface antibodies and washed into FoxP3-Fix/Perm buffer (eBioscience) at 4°C overnight. Samples were washed with FoxP3 Permwash and stained for intracellular antibodies. Cells were fixed in 1.6% formaldehyde solution for 1 hour prior to Iridium intercalation in Maxpar Fix/Perm Buffer (31.25 nM). Two additional water washes were performed immediately before running on the mass cytometer and samples were resuspended in 0.1X EQ four-element calibration beads (Fluidigm) at a concentration of 5×10^5^/mL. Samples were acquired on a Helios mass cytometer (Fluidigm) by the UNC mass cytometry core which is funded by the University Cancer Research Fund (UCRF) and UNC Cancer Center Core Support Grant #P30CA016086.

CYTOF analysis: FCS files were uploaded to the Astrolabe Cytometry Platform (Astrolabe Diagnostics) where transformation, debarcoding, cleaning, labeling, and unsupervised clustering was done. FCS files were normalized through median bead intensity and beads were removed prior to analysis. Single-cell data have been clustered using the *FlowSOM* R package (RRID:SCR_016899)^17^ and labeled using the Ek'Balam algorithm.^18^ The MDS map was generated using the *cmdscale* R function.^19^ Differential abundance analysis was done using the edgeR R package (RRID:SCR_012802),^20–22^ differential expression analysis was done using the *limma* R package (RRID:SCR_010943),^23 24^ and cell subset definitions^25 26^ follow published methods. The clusters were further analyzed using the Matthews Correlation Coefficient (MCC) to identify any additional changes in cytokine production.^17^ Cluster labeling, method implementation, differential abundance, differential expression, and visualization were done through the Astrolabe Cytometry Platform (Astrolabe Diagnostics, Inc.).

### Anti-CEA antibody response by ELISA

Patient sera were collected at weeks 0, 3, 6, 9, and 12. 96-well plates were coated with whole CEA protein (100 ng/well) and incubated with 100 µL of serum in duplicate serially diluted 1:25 to 1:1600. Titers were defined as the highest dilution such that the mean absorbance was equal to twice the negative control.

### Analysis of antivector responses with a VRP neutralization assay

To determine antivector responses, antibodies to VRP were measured using a modified neutralization assay previously described.^27^ VRP expressing HER2 was mixed with serial dilutions of patient sera and then added to Vero cells (RRID:CVCL_0059). The number of cells expressing HER2 for each serum dilution was determined by flow cytometry.

### Statistical analyses

For clinical studies, descriptive statistics are presented. Relapse-free survival (RFS) was defined as the time from surgery to disease recurrence or death from any cause, whichever came first. For patients with stage III cancer, OS was defined from the time of surgery until last follow-up or death due to any cause. For patients with stage IV cancer, the starting date for OS was the date of study enrollment. RFS and OS were calculated using the Kaplan-Meier method. Radiographic response was determined according to RECIST criteria 1.1. A paired Student’s t test was used to determine differences prevaccination and postvaccination. Data were analyzed using SAS software V.9.4 (Copyright 2016 SAS Institute; RRID:SCR_008567) and RStudio (R V.3.6.1).

## Results

### Long-term survival in patients with stage IV cancer treated with VRP-CEA(6D)

**Figure 1:**
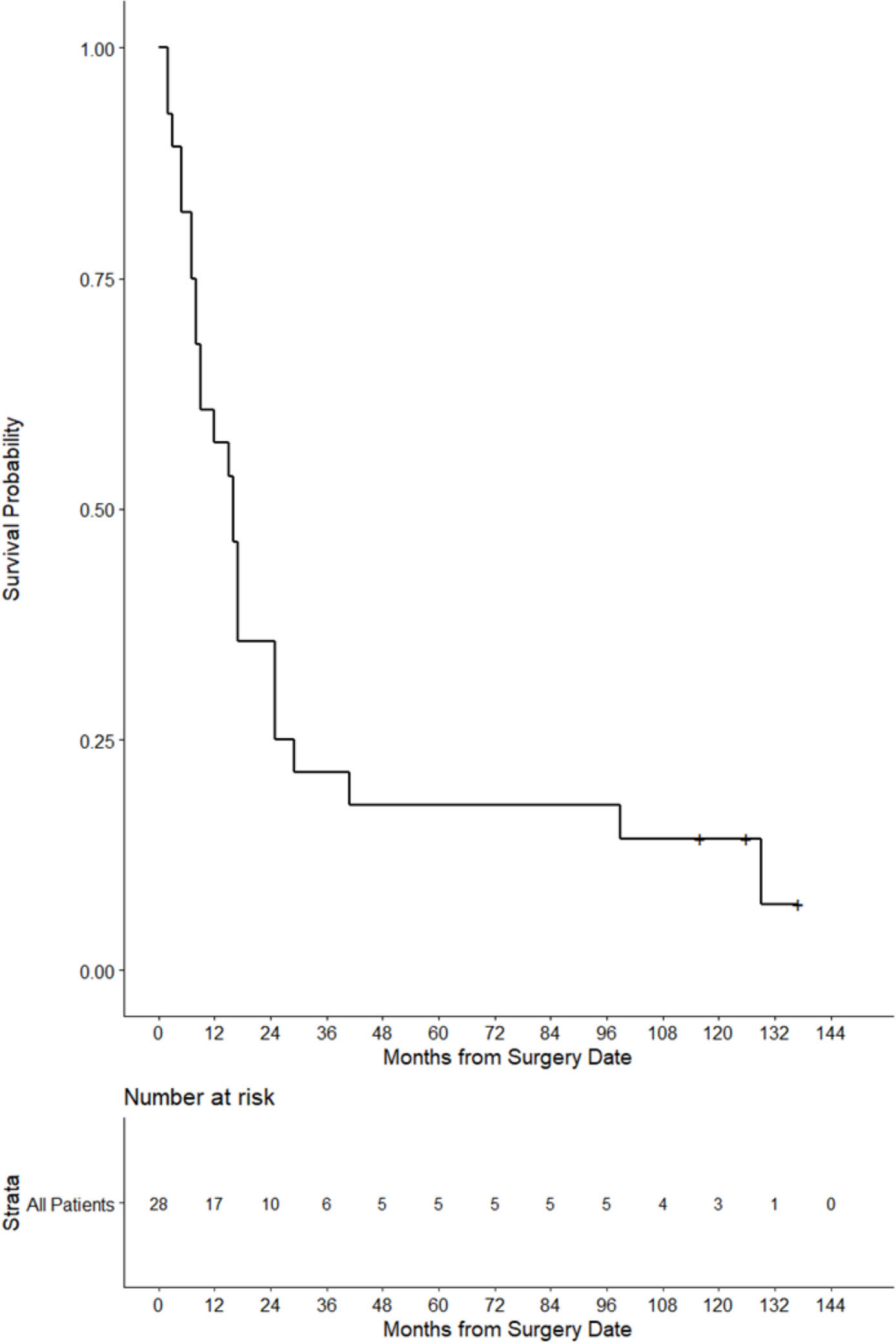
Overall survival of all 28 patients with stage IV malignancies enrolled in the phase I study of VRP-CEA. CEA, carcinoembryonic antigen; VRP, virus-like replicon particle.

In the prior clinical trial of VRP-CEA(6D) enrolling patients with metastatic malignancies (predominantly colon cancer), we observed vaccine-induced adaptive immunity and reported longer survival for those with CEA-specific T cell responses (details and demographics previously published).^12^ We now update their survival, with median follow-up of 10.9 years; 95% CI (9.6 to 11.4) with 10-year survival of 0.14; 95 % CI (0.04 to 0.29). Three of 28 (3/28) patients were alive at 9.6, 10.5, and 11.4 years, respectively, from study enrollment (figure 1). These three individuals had previously treated metastatic cancer, but minimal or no evidence of disease at the time of enrollment, suggesting that activity of the vaccine may be greater in those with the least tumor-induced immunosuppression. We, therefore, designed a pilot study to assess the immunogenicity and clinical activity of VRP-CEA(6D) in a group of patients with no evidence of disease but significant risk of recurrence, those with stage III colon cancer who had their primary disease resected and completed adjuvant chemotherapy.

### Patients with stage III cancer treated with VRP-CEA(6D)—patient demographics and treatment

Patients (n=12) with stage III colon cancer were subsequently enrolled onto this study at one center from January 2014 to February 2017 when the study was discontinued due to slow accrual. Demographics are provided in table 1. The majority (67%) had colon cancer stage IIIB and all had received standard adjuvant chemotherapy with FOLFOX or CAPOX with the exception of one patient who had complications from their first cycle of fluorouracil and received irinotecan plus oxaliplatin afterwards. Two patients with rectal cancer had received preoperative radiotherapy. As expected, due to the postoperative recovery time and the typical 6 months of adjuvant therapy, patients enrolled a median of 10.3 months from their surgery. All patients received the planned four doses of VRP-CEA(6D). There were no toxicities referable to the immunizations, but all reported adverse events are listed in online supplemental table 2.

10.1136/jitc-2020-001662.supp2 Supplementary data

**Table 1:** Demographics for stage III VRP-CEA vaccine study

Characteristic	N (%)
Median age at study entry	53 (IQR 43–62)
Gender (female/male)	8 (67)/4 (33)
Race (Caucasian/non-Caucasian)	11 (92)/1 (8)
Site (colon/rectum)	10 (83)/2 (17)
Stage	
IIIA	1 (8)
IIIB	8 (67)
IIIC	3 (25)
Adjuvant therapy	
Chemotherapy	10 (83)
Chemotherapy and radiotherapy	2 (17)
Time from surgery to study enrollment (months)	10.5 (IQR 8.8–11.3)

CEA, carcinoembryonic antigen; VRP, virus-like replicon particle.

### Induction of T cell responses by VRP-CEA(6D)

In our previous studies of vaccination with VRP-based vectors, we used IFNγ-ELISPOT to evaluate the T cell responses to CEA following vaccination.^12 28^ In the current study, we evaluated CEA-specific IFNγ-ELISPOT responses in the first six patients and saw an increase in IFNγ responses in all patients in at least one timepoint tested postvaccination (online supplemental figure 1). The highest response postvaccine is shown for each patient. While this is a valuable tool, it is more difficult to determine small changes in antigen-specific T cell responsiveness and does not give information about T cell subsets responding to the vaccinations.

10.1136/jitc-2020-001662.supp3 Supplementary data

**Figure 2:**
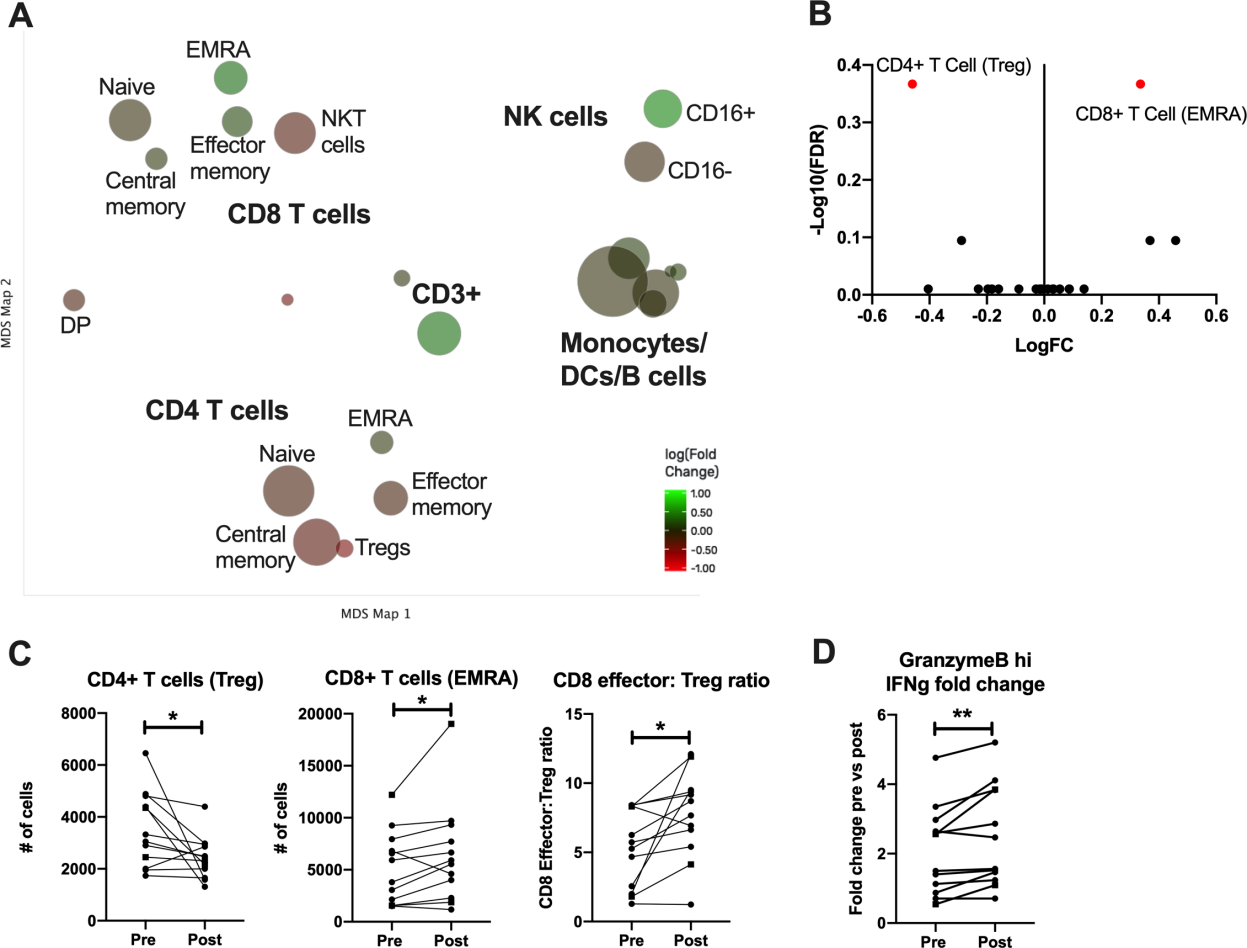
CYTOF analysis of PBMCs restimulated with CEA from patients prevaccination and postvaccination. (A) Cells were clustered and visualized using MDS. Each cluster is colored based on magnitude of fold change from the prevaccine to postvaccine sample after restimulation with TRICOM-CEA. (B) Volcano plot of identified cell clusters from (A), showing those with a significant p value in red. (C) Number of cells in each indicated cluster or the ratio of activated CD8 T cells to Tregs paired for each patient prevaccination and postvaccination. Samples from patients with colorectal cancer are shown as dashed lines with square symbols. (D) The fold change in IFN-γ production by granzyme B hi CD8 T cells post-TRICOM-CEA restimulation paired for each patient prevaccination and postvaccination. *P<0.05 **p<0.01. CEA, carcinoembryonic antigen; CYTOF, cytometry time of flight; MDS, multidimensional scaling; PBMS, peripheral blood mononuclear cell; VRP, virus-like replicon particle.

In order to characterize more comprehensively the induction of systemic T cell responses postvaccination, peripheral blood mononuclear cells (PBMCs) obtained before and after immunization were evaluated by multiparameter mass cytometry (CyTOF) following stimulation with a different CEA-encoding viral vector than used for vaccination (rF-CEA(6D)-TRICOM). The time postvaccination was dependent on PBMC availability, but all data are shown with paired prevaccine and postvaccine measurements for each patient. Cells were clustered using cell surface markers and expression of effector molecules prevaccination and postvaccination within these clusters was analyzed (figure 2A). Cell types were grouped into canonical subsets based on a gating hierarchy that corresponds to traditional gating used for flow cytometric analysis of surface markers. This process is automated to limit the introduction of bias that can occur when determining gates manually. To visualize the large amount of data that are generated by this CYTOF staining panel, we used a Multidimensional Scaling (MDS) map. Each bubble in this map represents a cell subset that was identified and analyzed and the size of that bubble is determined by the median frequency of cells contained in that bubble across all samples analyzed. We then compared the frequency of each identified cell type postvaccination to identify how these immune subsets were altered by the vaccine. Each bubble is colored based on the magnitude of fold change from the prevaccine to postvaccine sample after restimulation with TRICOM-CEA. A volcano plot summarizes the changes in subset frequency and the statistical significance of each change (figure 2B). We can see here that the only two cell subsets that are significantly changed postvaccination are CD8+T_EMRA_, which increase postvaccination, and Tregs which decrease (shown in red). These changes are shown for each individual patient as well (figure 2C). We observed that CD8+T_EM_ (and in particular the terminally differentiated effector memory cells CD8 T_EMRA_) were increased in 10/12 (83.3%) patients and Tregs were decreased in 10/12 (83.3%) patients following the immunizations, with both parameters changing in 8/12 (66.7%) patients. Analysis of both parameters taken together shows that the CD8 T_EMRA_:Treg ratio increased in 10/12 (83.3%) patients (figure 2C).

In addition to the cell surface marker based gating analysis, we used an unsupervised clustering algorithm to identify any subsets of all of the previously identified cell types that had statistically significant differential expression of cytokines following stimulation with CEA. As a result, we show that CD8+granzyme B+T_CM_ cells generating IFNγ were increased (in 9/12 (75%)). No other cell type was significantly changed by immunization. These data indicate that an activated, cytolytic T cell population specific for CEA is induced by the VRP-CEA without resulting in an enhanced immunosuppressive population.

### Induction of antibody responses by VRP-CEA(6D)

**Figure 3:**
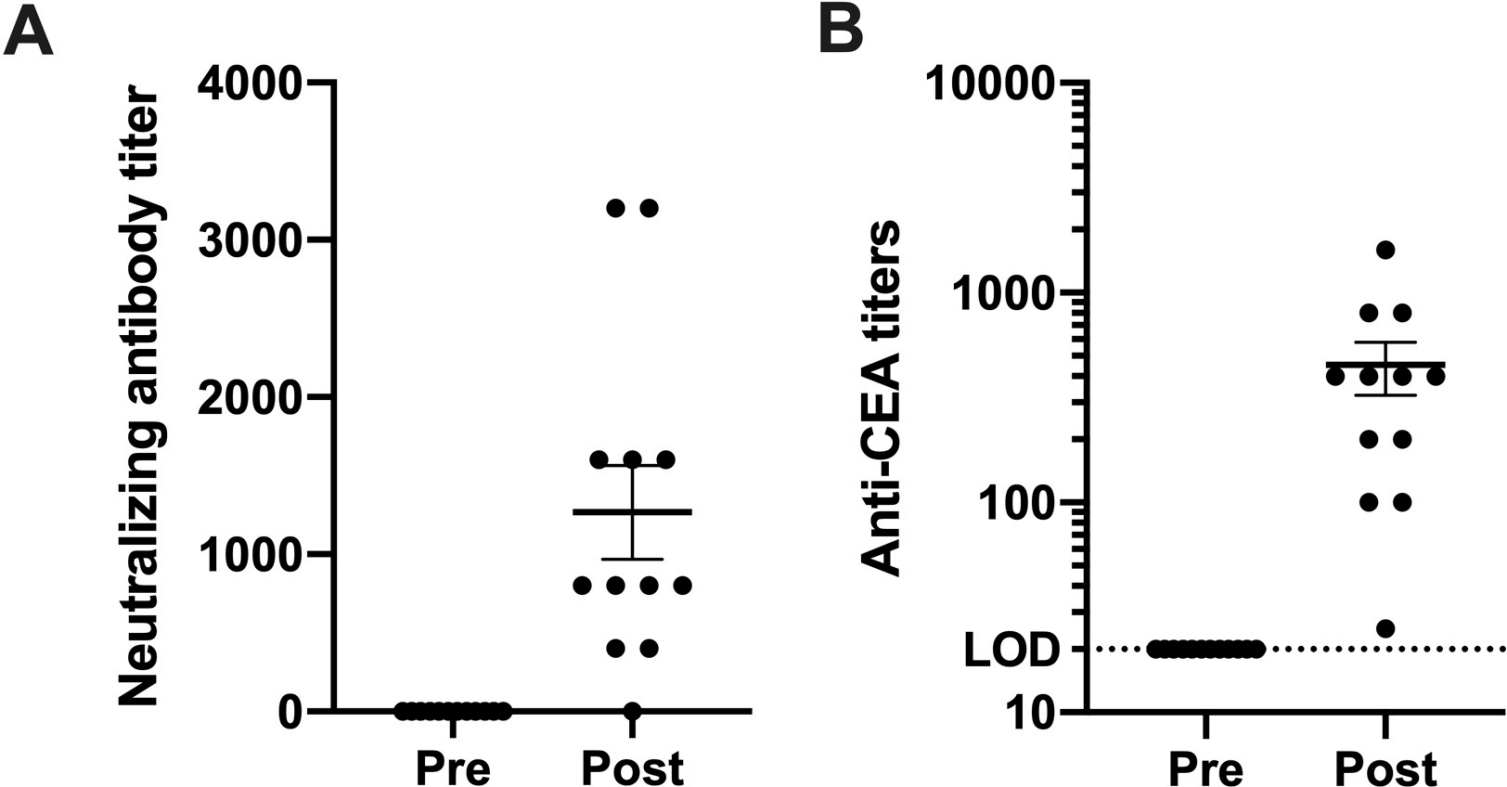
VPR neutralizing antibody titer before initiating immunizations and after immunizations were completed. (A) Patient sera were analyzed before and after VRP-CEA in anti-VRP neutralization assay. The endpoint titer was defined as the last serum dilution at which there was at least 80% reduction in the number of VRP positive cells compared with control wells. (B) Patient sera were analyzed for anti-VRP antibodies on weeks 0, 3, 6, 9, and 12. The antibody titer is presented for prevaccination and the highest postvaccination response. Values shown as mean±SEM. CEA, carcinoembryonic antigen; VRP, virus-like replicon particle.

In our previous VRP-CEA study in patients with stage IV cancer,^12^ although viral replicon particle (VRP)-induced neutralizing antibodies were detected, we were nonetheless able to immunize repeatedly, increasing the humoral immune response. In the present study, neutralizing antibodies against VRP were induced in all but one patient after immunization (figure 3A). As before, despite these neutralizing antibodies, VRP-CEA activated CEA-specific antibodies with titers above baseline were noted in all patients (figure 3B). Consistent with our prior reports, these data suggest that the neutralizing antibodies, common to most viral vector platforms, do not impair VRP-CEA immunogenicity. As with the ELISPOT responses, the CEA specific antibody responses in patients with stage III cancer were significantly higher than those seen in patients with stage IV cancer. The anti-CEA titers in patients with stage IV cancer averaged a maximum titer of 80,^12^ while patients with stage III cancer in this cohort had an average titer of nearly 500 (figure 3B).

### Clinical outcomes

**Figure 4:**
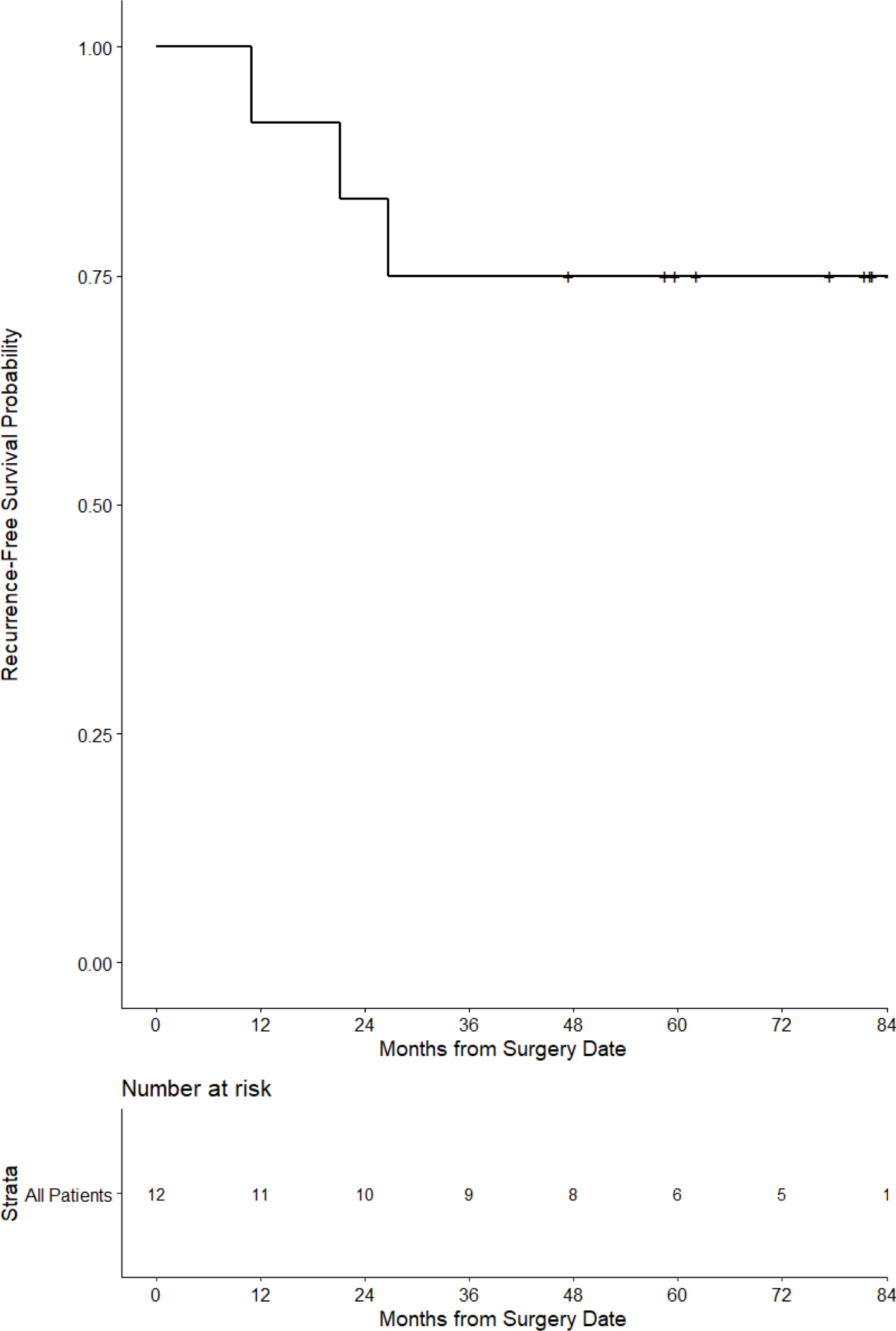
Progression-free survival for the stage III patients immunized with VRP-CEA. CEA, carcinoembryonic antigen; VRP, virus-like replicon particle.

The clinical status of all participants was ascertained periodically following completion of treatment. At a median follow-up of 60 months (5.8 years), all patients remained alive and 3/12 (25%) had recurrent disease (figure 4).

## Discussion

Many malignancies, despite resection and standard oncologic therapies, continue to have high risks of relapse. Stage III colon cancer, prior to the broad implementation of systemic therapy, was frequently lethal due to the emergence of recurrent disease in a majority of patients. The IDEA collaborative^29^ reported recently that the 5-year OS rate was 82.4% and 82.8% for 3 months and 6 months of postoperative adjuvant chemotherapy, respectively. The 5-year DFS rates were 69.1% and 70.8%, respectively. This risk of recurrence in a scenario with minimal tumor burden to cause immunosuppression represents an ideal scenario for testing a cancer vaccine. Having demonstrated the immunogenicity of VRP-CEA in the presence of elevated levels of regulatory T cells in patients with advanced colorectal cancer,^12^ despite vector-induced neutralizing antibodies, we noted the long-term survival of a subset (3/28, 11%) of vaccinated patients with stage IV cancer. To determine if immune responses and clinical benefit would be enhanced in patients with less tumor burden, we performed this pilot study to assess the immunogenicity and estimate clinical results of VRP-CEA vaccination in patients with stage III colorectal cancer. The immunizations were well tolerated, an important point for a population of patients often left with chronic neuropathy from prior therapy and for whom the field is attempting to reduce the amount of therapy administered. At a median follow-up of 5.8 years, survival was 100% and only 3/12 (25%) had experienced recurrent disease, both of which are not inconsistent with the outcome from the IDEA collaborative.^29^ Anti-CEA antibody levels above baseline were detected in all patients and the majority of patients experienced an increase in systemic CEA-specific T cell responses, indicating a highly functional vaccine.

An important strength of this study was the use of CyTOF to measure the immune response in the peripheral blood following immunization. In our prior study with VRP-CEA in patients with stage IV cancer, we exclusively used ELISPOT analysis to characterize the T cell response; however, this provides a narrow description of immunogenicity and fails to completely capture the magnitude of T cells capable of responding to CEA before and after immunization. We recently reported on the use of CyTOF analysis to describe the changes occurring in peripheral blood immune cells after vaccination with a different VRP-based vaccine encoding the tumor antigen HER2 (VRP-HER2).^28^ In that study, we also observed the expansion of an antigen-specific, cytolytic CD8 T cell population, suggesting that CyTOF may be a preferred approach for assessing immunogenicity of cancer vaccines. In the current study, CyTOF allowed us to determine that the terminally differentiated effector memory cells CD8+T_EMRA_ were increased and Tregs were decreased in most patients following the immunizations, with the result that the CD8 T_EMRA_:Treg ratio increased, an important indicator of a favorable environment for antitumor immune responses. Because our prior study with VRP-CEA did not use CyTOF analysis, we cannot directly compare the immunogenicity of this vector in patients with stage III versus stage IV colon cancer; however, we did perform an IFNγ-ELISPOT in a limited number of patients in the current study and observed much higher magnitudes of CEA-specific T cell responses compared with those seen in the stage IV study.^12^ The highest responses seen in patients with stage IV cancer averaged approximately 40 IFN-γ producing cells per 10^6^ cells while patients with stage III cancer in this study had an average of approximately 150 IFN-γ producing cells (online supplemental figure 1).

Because the OS and DFS of the vaccinated subjects were consistent with studies of patients who have received adjuvant therapy for stage III colon cancer, we were interested in determining whether any immune changes correlated with improved DFS. Due to the small number of recurrences, we were unable to make this assessment; however, the impact of immunotherapy may be greatest on OS which may take longer to demonstrate. In a clinical trial of a poxvector-based, CEA-targeting vaccine evaluated in patients with resected colorectal metastases, we found that OS but not recurrence-free survival was improved with the immunizations compared with unvaccinated patients.^15^ We will continue to follow participants in the current study to determine whether OS eventually does correlate with an immune biomarker.

Subsequent studies will attempt to enhance the potency of the induced T cell and antibody responses. One strategy could involve immunization in the neoadjuvant setting prior to surgical resection, a concept that has preliminarily demonstrated benefit for other forms of immunotherapy (eg, ICB) in other malignancies.^30 31^ Another strategy could include a combination of the VRP-CEA with ICB. We recently reported that in mouse models of colon cancer, the combination of a viral-CEA vaccine with anti-PD-1 antibody resulted in greater antitumor activity and immune responses compared with vaccination against CEA alone.^32^

We conclude that immunization of patients with colon cancer with a viral replicon-based cancer vaccine induces antigen-specific effector T cells while decreasing Tregs, a feature of effective antitumor immune responses. Patients with minimal residual disease achieved surgically appear to have the greatest benefit from this vaccine. Future studies will test novel dosing schedules and combinations with ICB.
