# Supplementary material for: Long-term survival of patients with stage III colon cancer treated with VRP-CEA(6D), an alphavirus vector that increases the CD8+ effector memory T cell to Treg ratio
Source: J Immunother Cancer. 2020 Nov 11;8(2):e001662. doi: 10.1136/jitc-2020-001662 (PMC7661359; doi:10.1136/jitc-2020-001662)
Supplement: Supplementary data [file jitc-2020-001662supp001.pdf]

Supplementary table 1. List of antibodies used for CYTOF panel.

Target	Clone	Metal	product_id	catalog
CD45	HI30	89Y	3089003B	FDM
CD45	HI30	115In	0	Lederer
CD14	M5E2	151Eu	3151009B	FDM
CD3	UCHT1	154Sm	3154003B	FDM
CD161	HP-3G10	159Tb	3159004B	FDM
CD56 (NCAM)	B159	163Dy	3163007B	FDM
CD20	2H7	171Yb	3171012B	FDM
CD16	3G8	209Bi	3209002B	FDM
CD11a	HI111	142Nd	3142006B	FDM
CD45RA	HI100	153Eu	3153001B	FDM
CD45RO	UCHL1	164Dy	3164007B	FDM
CD8a	SK1	168Er	3168002B	FDM
CD4	RPA-T4	176Yb	3176010B	FDM
HLA-DR	L243	147Sm	0	Lederer
CD69	FN50	144Nd	3144018B	FDM
CD127 (IL-7Ra)	A019D5	149Sm	3149011B	FDM
CD223 (Lag3)	LAG-3	165Ho	3165037B	FDM
CD27	O323	167Er	3167002B	FDM
CD25 (IL-2R)	2A3	169Tm	3169003B	FDM
CD44	BJ18	141Pr	0	Lederer
CD279 (PD-1)	EH12.2H7	143Nd	0	Lederer
CD197 (CCR7) Biotin	G043H7	biotin	353240	Biolegend
TNFa	Mab11	152Sm	3152002B	FDM
IL-2	MQ1-17H12	158Gd	3158007B	FDM
FoxP3	PCH101	162Dy	3162011A	FDM
Granzyme B	GB11	173Yb	3173006B	FDM
Perforin	B-D48	175Lu	3175004B	FDM
IFNg	B27	155Gd	0	Lederer
CD152 (CTLA-4) FITC	14D3	FITC	11-1529-42	ThermoFisher
Anti-FITC	FIT-22	160Gd	3160011B	FDM
Anti-Biotin	1D4-C5	150Nd	3150008B	FDM
