# Supplementary material for: Long-term survival of patients with stage III colon cancer treated with VRP-CEA(6D), an alphavirus vector that increases the CD8+ effector memory T cell to Treg ratio
Source: J Immunother Cancer. 2020 Nov 11;8(2):e001662. doi: 10.1136/jitc-2020-001662 (PMC7661359; doi:10.1136/jitc-2020-001662)
Supplement: Supplementary data [file jitc-2020-001662supp002.pdf]

Supplementary Table 2. All reported adverse events

Grade of Adverse Event	1- Mild		2- Mod		3-Severe		4-LifeThr		5-Lethal		Total
	n	(%)	n	(%)	n	(%)	n	(%)	n	(%)	N
**Non-Hematologic Adverse Events**											
Eye disorders - Other, Specify	1	8%	0	0%	0	0%	0	0%	0	0%	12
General disorders and administration site conditions - Other, Specify	1	8%	0	0%	0	0%	0	0%	0	0%	12
**Endocrine disorders**											
Hyperthyroidism	0	0%	1	8%	0	0%	0	0%	0	0%	12
**Gastrointestinal disorders**											
Abdominal pain	1	8%	1	8%	0	0%	0	0%	0	0%	12
Constipation	1	8%	1	8%	0	0%	0	0%	0	0%	12
Diarrhea	1	8%	2	17%	0	0%	0	0%	0	0%	12
Rectal hemorrhage	1	8%	0	0%	0	0%	0	0%	0	0%	12
**General disorders and administration site conditions**											
Fatigue	0	0%	1	8%	0	0%	0	0%	0	0%	12
Fever	2	17%	0	0%	0	0%	0	0%	0	0%	12
Flu like symptoms	1	8%	0	0%	0	0%	0	0%	0	0%	12
Injection site reaction	3	25%	0	0%	0	0%	0	0%	0	0%	12
Pain	2	17%	0	0%	0	0%	0	0%	0	0%	12
**Infections and infestations**											
Mucosal infection	0	0%	1	8%	0	0%	0	0%	0	0%	12
Skin infection	1	8%	0	0%	0	0%	0	0%	0	0%	12
Upper respiratory infection	1	8%	0	0%	0	0%	0	0%	0	0%	12
**Investigations**											
Blood bilirubin increased	1	8%	0	0%	0	0%	0	0%	0	0%	12
**Musculoskeletal and connective tissue disorders**											
Arthralgia	1	8%	0	0%	0	0%	0	0%	0	0%	12
**Nervous system disorders**											
Headache	0	0%	1	8%	0	0%	0	0%	0	0%	12
**Respiratory, thoracic, and mediastinal disorders**											
Cough	2	8%	0	0%	0	0%	0	0%	0	0%	12
Pleuritic pain	1	8%	0	0%	0	0%	0	0%	0	0%	12
**Skin and subcutaneous tissue disorders**											
Scalp pain	0	0%	1	8%	0	0%	0	0%	0	0%	12
**Summary**											
**Maximum Non-Hematologic AE**	**6**	**50%**	**5**	**42%**	**0**	**0%**	**0**	**0%**	**0**	**0%**	**12**
**Maximum Overall AE**	**6**	**50%**	**5**	**42%**	**0**	**0%**	**0**	**0%**	**0**	**0%**	**12**

BMJ Publishing Group Limited (BMJ) disclaims all liability and responsibility arising from any reliance placed on this supplemental material which has been supplied by the author(s)
