# Supplementary material for: Long-term survival of patients with stage III colon cancer treated with VRP-CEA(6D), an alphavirus vector that increases the CD8+ effector memory T cell to Treg ratio
Source: J Immunother Cancer. 2020 Nov 11;8(2):e001662. doi: 10.1136/jitc-2020-001662 (PMC7661359; doi:10.1136/jitc-2020-001662)
Supplement: Supplementary data [file jitc-2020-001662supp003.pdf]

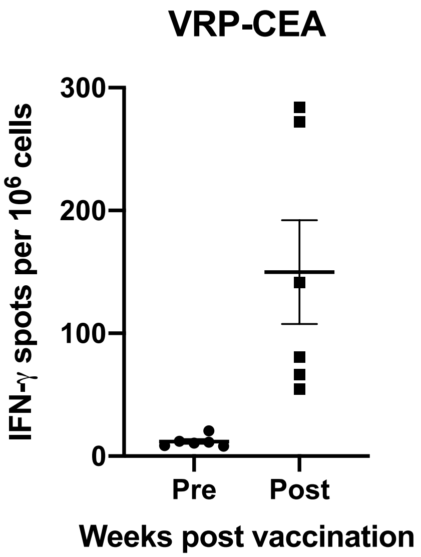

Supplemental figure 1: CEA-specific ELISPOT responses in the first 6 patients. Patient sera or PBMCs were analyzed before and after VRP-CEA(6D) for each immunization by VRP-CEA and TRICOM-CEA IFN-γ ELISpot for the first six patients on study. Patient PBMCs were stimulated with VRP-CEA or TRICOM-CEA (MOI 10) in an ELISpot assay (previously described). The number of IFN-γ–producing cells per 10^6 PBMCs is presented as mean ± SEM.
